# Prophylactic Bilateral Salpingo-oophorectomy in BRCA2 Mutation with Incidental Finding of Serous Tubal Intraepithelial Carcinoma (STIC) and Subsequent Diagnosis of Primary Peritoneal Carcinoma (PPC): A Case Report and Review of Current Literature

**DOI:** 10.7759/cureus.9301

**Published:** 2020-07-20

**Authors:** Konstantinos Palaiologos, Ahmed Ellaboudy, Mohammed Abdullah, Seema Karan, Arabinda Saha

**Affiliations:** 1 Obstetrics and Gynecology, Diana Princess of Wales Hospital, Grimsby, GBR; 2 Radiology, Scunthorpe General Hospital, Scunthorpe, GBR

**Keywords:** risk reducing salpingo-oophorectomy, brca gene mutation, prophylactic bilateral salpingo-oophorectomy, serous tubal intraepithelial carcinoma, peritoneal carcinoma

## Abstract

A major effort to prevent serous cancer in genetically susceptible women with breast cancer susceptibility gene (BRCA) mutations has recently introduced the practice of risk-reducing prophylactic salpingo-oophorectomy. A small number of those who undergo prophylactic salpingo-oophorectomy will be found to have occult carcinomas. The majority of these appear to originate in the fallopian tube, reinforcing the theory that a significant proportion of high-grade serous carcinoma pelvic tumours have a fimbrial origin. In addition to this, histopathological and molecular biological characteristics suggest that among other serous carcinomas, fallopian tube serous carcinoma and primary peritoneal serous carcinoma really represent one entity. We present a case with breast cancer susceptibility gene 2 (BRCA2) mutation that was found to have serous tubal intraepithelial carcinoma (STIC) following prophylactic salpingo-oophorectomy. Subsequently, she was diagnosed with advanced primary peritoneal carcinoma. This prompted our team to reflect upon the case, review the current literature and recommend a rigorous preoperative assessment and meticulous intraoperative examination for prevention and early detection of high grade serous pelvic carcinomas.

## Introduction

Breast cancer susceptibility gene (BRCA) mutation was discovered in the 90s. It all started when Mary-Claire King’s genetic studies indicated a locus on chromosome 17p for a putative susceptibility gene. Shortly thereafter, the identification and cloning of the breast cancer susceptibility genes 1 and 2 (BRCA1 and BRCA2) took place [1]. Since then, there has been a dramatic breakthrough in the prevention of breast and ovarian cancers. According to the recent literature, women with BRCA1 mutation have a 72% lifetime risk of developing breast cancer and 44% of ovarian cancer, whereas, BRCA2 gene mutation has lifetime risks of 69% and 17%, respectively [2]. Regarding prophylaxis for breast cancer, women carriers of the gene mutation are either offered annual screening with mammograms until 70 and after that, every three years, or they can undergo prophylactic bilateral mastectomy with reconstructive surgery. Unfortunately, there is no screening test proven to be effective in detecting ovarian cancer at early stages. In addition, the tools we have so far such as cancer antigen 125 (CA125), transvaginal ultrasound scan, and magnetic resonance imaging have limited diagnostic value. Therefore, the current recommendation is to undergo risk-reducing bilateral salpingo-oophorectomy (RRSO) usually utilizing the laparoscopic approach. We present a case of a BRCA gene mutation carrier who following RRSO was found to have focal serous tubal intraepithelial carcinoma (STIC) and subsequently advanced primary peritoneal malignancy.

## Case presentation

Our patient was a 75-year-old white woman who was a carrier of the BRCA2 gene mutation. She presented to her primary care doctor with a family history of three of her daughters having been diagnosed with breast cancer. All of them were BRCA2 positive. She had no other issue otherwise. Consequently, she underwent genetic testing for BRCA gene mutations which was reported positive for BRCA2 gene mutation. The patient was then informed about the increased risks of breast and ovarian cancers. Regarding the increased risk for breast cancer, the patient was informed about the options of having screening every three years along with prophylactic treatment with tamoxifen or anastrozole or undergoing a prophylactic bilateral mastectomy with reconstruction. She chose to have screening mammogram every three years. After thorough discussion and counselling in the clinic, the patient was informed that the best option to minimize the risk of ovarian cancer would be to have the ovaries and fallopian tubes removed laparoscopically.

The patient was reviewed in the clinic prior to her elective procedure. Her past medical history consisted of hypertension, osteoarthritis, seborrheic dermatitis, and hepatitis A infection in 1970. Her past surgical history included laparoscopic sterilization and left knee replacement. She was on lansoprazole, amlodipine, and antihistaminic medication and she had no known allergy. She had four previous normal vaginal deliveries and she was up to date with her cervical screening, with the last one being negative. Her body mass index (BMI) was 32 and the routine blood results including urea and electrolytes, full blood count and coagulation profile were all within normal limits.

She had an elective laparoscopic bilateral salpingo-oophorectomy without any intraoperative complication. A small retroverted uterus was noted and both tubes and ovaries looked normal. The course of the uterer was identified on both sides. The left ovary was adherent to the left pelvic side wall and was mobilised before removal. An electrothermal bipolar tissue sealing device was used and the specimen was delivered with a laparoscopic bag. The tubes and ovaries were sent in the same container for histological examination. The blood loss was minimal. The patient had an uneventful recovery and was discharged home the same day.

**Figure 1:**
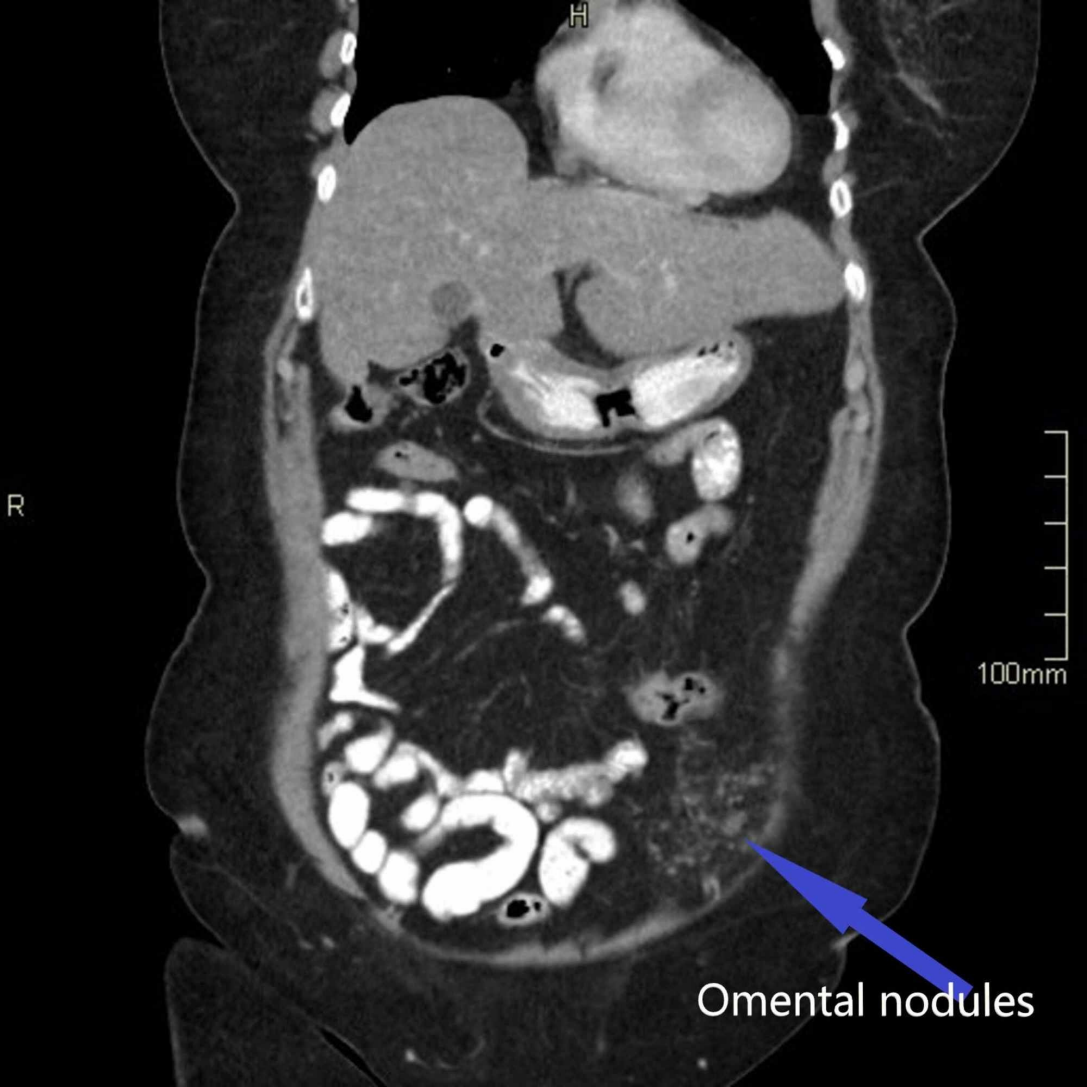
Coronal view from CT scan

**Figure 2:**
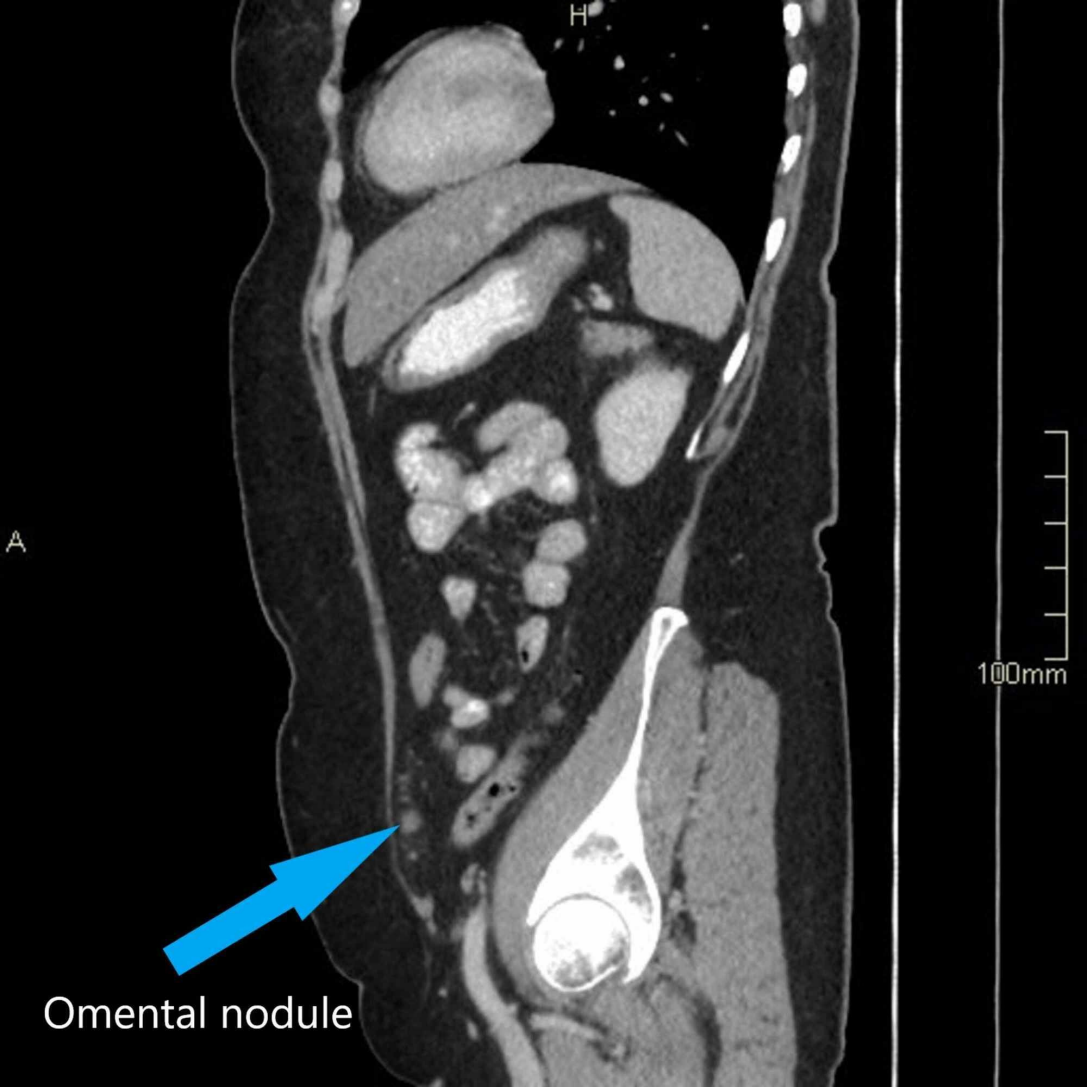
Sagittal view from CT scan

**Figure 3:**
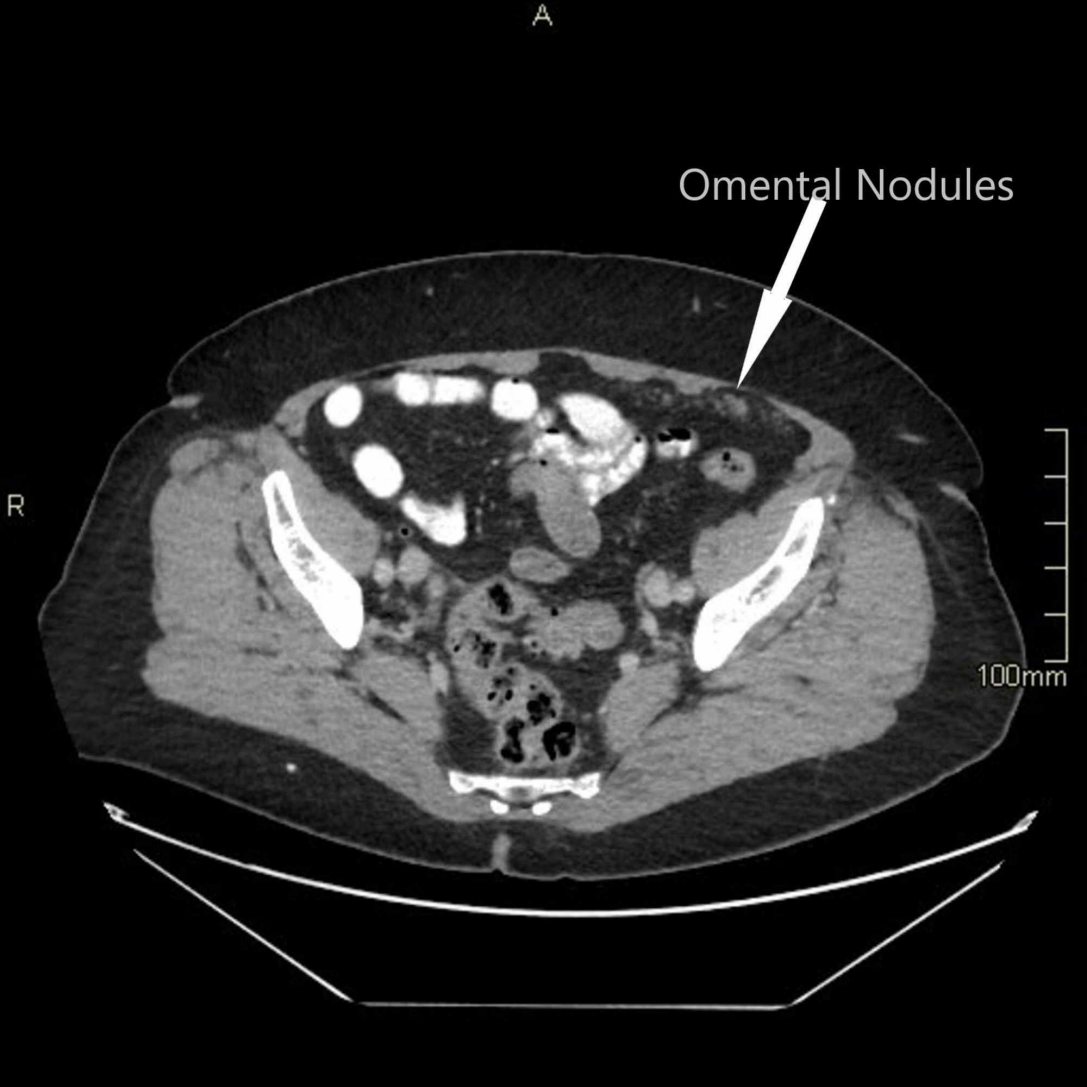
Axial view from CT scan

The histopathologist reported that one of the fallopian tubes showed elements of a STIC with negative staining for p53. The Ki67 index was at least 30% but with no evidence of invasive malignancy. The other tube and both ovaries were normal. Following this, the patient’s case and the histology results were discussed in the regional multidisciplinary meeting (MDT). A decision was made for the patient to have a CT chest/abdomen/pelvis followed by completion surgery. The CT thorax, abdomen, and pelvis showed tiny omental nodules in the left anterior hemipelvis which were suspicious of metastasis (Figures 1-3). The soft tissue stranding in the parametrium was thought to be post-surgical changes. Following a discussion in the MDT, she was seen in the centre gynae-oncology unit and was offered further surgical management.

She had a total abdominal hysterectomy, supracolic omentectomy, appendicectomy, peritonectomy with multiple peritoneal biopsies and resection of the disease from the pouch of Douglas. The intraoperative findings were consistent with advanced disease. The diaphragmatic surfaces were normal. The omentum appeared thickened but the lesser sac of the omentum was normal. There were small 5-10 mm miliary deposits affecting both left and right paracolic gutters, Gerota’s fascia, pouch of Douglas, and recto-sigmoid with further smaller military deposits on small bowel mesentery.

The histology confirmed widespread high-grade serous carcinoma amounting to primary tubal malignancy International Federation of Gynecology and Obstetrics (FIGO) stage 3c. The case was discussed again in the MDT and the patient was referred to the medical oncologist for consideration of adjuvant chemotherapy. She was commenced on adjuvant cytotoxic chemotherapy with paclitaxel and carboplatin. In view of the Covid-19 pandemic, primary prophylaxis of granulocyte-colony stimulating factor (GCSF) was offered. The plan was to commence olaparib maintenance following completion of the six chemotherapy cycles.

## Discussion

At present, the number of women having genetic testing is on the rise. As genetic testing for BRCA genes is more available now, health professionals are aware of the risk factors (refer Appendices).

As a result, more women are diagnosed with BRCA gene mutations and consequently, the number of RRSO for BRCA gene mutations is going up. For BRCA1 carriers RRSO is recommended before the age of 40 and for BRCA2 carriers RRSO is recommended before the age of 45 or in any case when their family is complete [3]. However, even after surgery, there is a risk of 1%-4.3% of developing peritoneal carcinoma [4]. A woman who declines salpingo-oophorectomy can undergo screening with the use of serum measurement of CA125 and transvaginal ultrasonography every six to twelve months, starting at age 30 to 35 years or five to ten years before the earliest diagnosis of ovarian cancer in the family [5].

RRSO in BRCA gene mutations has decreased the risk of ovarian cancer significantly. However, the site of origin of pelvic high-grade serous carcinoma (HGPSC) has been the subject of debate for 60 years. Several emerging evidence have suggested that some high-grade serous carcinomas of the pelvis originate from the fimbrial ends of the fallopian tubes [6,7]. This was previously considered to be originating from the ovaries. STIC is a lesion limited to the fallopian tube epithelium that is considered to be the precursor to extrauterine (pelvic) high-grade serous carcinoma. Multiple studies indicate that identification of STIC can help us detect HGPSC before it presents at an advanced stage [8]. This discovery that many pelvic serous cancers originate in the fallopian tubes raises the question of whether bilateral salpingectomy with delayed oophorectomy may be an option for premenopausal women who want to delay iatrogenic menopause [8]. However, this is discouraged outside clinical trials as women would still be at risk of developing ovarian cancer [9].

Among women undergoing prophylactic bilateral salpingo-oophorectomy, STIC has been reported in up to 70% of specimens when a concurrent invasive cancer is present [10,11] and 2%-8% when invasive cancer is absent [4,12]. In the general population, STIC has been identified in 18%-71% of surgical pathology specimens removed for HGSC [7,13,14] and incidentally in less than 1% of women undergoing benign surgery [15,16]. Frequent detection of occult STIC among pathogenic BRCA1 or BRCA2 mutation carriers at prophylactic bilateral salpingo-oophorectomy prompted the hypothesis that many adnexal high-grade serous carcinomas (HGSCs) arise in the fallopian tube, rather the ovary, as previously presumed [17-19].

This case prompted constructive reflection in our local gynaecology team. Although there was no suspicion of pelvic or abdominal malignancy during the prophylactic bilateral salpingo-oophorectomy, a CT scan eleven weeks later was suggestive of metastatic disease. This was confirmed subsequently during laparotomy fifteen weeks after the first surgery. High-grade serous carcinomas are notoriously known for their aggressive pattern of disease behavior. It is also important to highlight that the laparoscopy is deficient as compared to laparotomy with regards to the palpation of the tissues.

Asymptomatic patients with BRCA gene mutation who opt-in for prophylactic surgery should be managed as a high-risk patient for potential malignancy until this is excluded. Therefore, we recommend a careful counselling of the patient and pre-operative assessment including CA125 and transvaginal pelvic ultrasound scan. If the CA125 is raised further magnetic resonance imaging might be required. Perioperatively, systematic and meticulous examination of the pelvis, abdominal peritoneum, pouch of Douglas, paracolic gutters, omentum, and diaphragm to check for signs of macroscopic disease is strongly advised. The clinician needs to be watchful for signs of ascites, peritoneal/omental disease or nodularity.

A very important point in RRSO is handling the samples. Samples should be handled by using the laparoscopic endoscopic bag to minimize the risk of spreading malignant cells in the abdominal cavity and port site metastasis. Moreover, the specimen should be labelled appropriately. Furthermore, different containers should be used for each side so that if any tube or ovary is found to be malignant, the information on laterality is available to help with further management. Last but not least, follow up of these cases is of great importance due to the overall increased risk of developing HGPSC in the long term.

Refer the Appendices - indications for BRCA testing as per NICE Clinical Guideline CG164 [20].

## Conclusions

The number of patients with BRCA mutation has increased lately due to health awareness and the availability of genetic testing. As a result, the number of RRSO surgery has greatly increased. Despite the routine nature of the RRSO operation, women with BRCA mutation are still at significant risk of concomitant high grade peritoneal serous carcinoma which is challenging to detect at early stages. A routine RRSO where there is occult cancer could potentially upstage the disease. We presented a case of a BRCA2 gene mutation carrier who initially opted for RRSO. The histology results reported STIC in one of the fallopian tubes. Subsequently, she had further investigations and went back to theatre for laparotomy, almost four months after the first surgery. The latter confirmed advanced peritoneal carcinoma. The case prompted our team to review the current literature and constructively reflect and re-evaluate the clinical management for routine RRSO for BRCA mutation carriers.

## Appendices

Indications for BRCA testing as per NICE Clinical Guideline CG164

People who meet the following referral criteria should be offered a referral to a specialist genetic clinic. 

At least the following female breast cancers only in the family

-            two first-degree or second-degree relatives diagnosed with breast cancer at younger than an average age of 50 years (at least one must be a first-degree relative)

or

-            three first-degree or second-degree relatives diagnosed with breast cancer at younger than an average age of 60 years (at least one must be a first-degree relative)

or

-            four relatives diagnosed with breast cancer at any age (at least one must be a first- degree relative). 

or

Families containing one relative with ovarian cancer at any age and, on the same side of the family

-            one first-degree relative (including the relative with ovarian cancer) or second- degree relative diagnosed with breast cancer at younger than age 50 years 

or

-            two first-degree or second-degree relatives diagnosed with breast cancer at younger than an average age of 60 years 

or

-            another ovarian cancer at any age. or

Families affected by bilateral cancer (each breast cancer has the same count value as one relative)

-            one first-degree relative with cancer diagnosed in both breasts at younger than an average age 50 years 

or

-            one first-degree or second-degree relative diagnosed with bilateral cancer and one first or second degree relative diagnosed with breast cancer at younger than an average age of 60 years.

or

Families containing male breast cancer at any age and, on the same side of the family, at least:

-            one first-degree or second-degree relative diagnosed with breast cancer at younger than age 50 years

or

-            two first-degree or second-degree relatives diagnosed with breast cancer at younger than an average age of 60 years. 

or

A formal risk assessment has given risk estimates of

-            a 10% or greater chance of a gene mutation being harboured in the family

or

-            a greater than 8% risk of developing breast cancer in the next 10 years or

-            a 30% or greater lifetime risk of developing breast cancer. 

Clinicians should seek further advice from a specialist genetics service for families containing any of the following, in addition to breast cancers:

-            triple negative breast cancer under the age of 40 years

-            Jewish ancestry 

-            sarcoma in a relative younger than 45 years

-            glioma or childhood adrenal cortical carcinomas

-            complicated patterns of multiple cancers at a young age 

-            very strong paternal history (four relatives diagnosed at younger than 60 years of age on the father’s side of the family).
